# Sweyjawbu expression is a predictor of ALK rearrangement status in lymphoma

**DOI:** 10.18632/oncotarget.13851

**Published:** 2016-12-10

**Authors:** Kai Xue, Xun Ye, Fang Liu, Qunlin Zhang, Qifeng Wang, Shan Huang, Jiachen Wang, YongMing Lu, Ye Guo, Xia Meng

**Affiliations:** ^1^ Department of Medical Oncology, Fudan University Shanghai Cancer Center, Shanghai, China; ^2^ Fudan University Shanghai Cancer Center, Institut Mérieux Lab, Fudan University Shanghai Cancer Center, Shanghai, China; ^3^ Department of Pathology, Fudan University Shanghai Cancer Center, Shanghai, China; ^4^ Department of Oncology, Shanghai Medical College, Fudan University, Shanghai, China; ^5^ Medical Device Development Department (MD3), bioMérieux Co., Ltd., Shanghai, China; ^6^ Department of Pathology, The First Affiliated Hospital of Soochow University, Suzhou, China

**Keywords:** sweyjawbu, ALK rearrangements, lymphoma

## Abstract

In recent years molecular subtyping has become an important tool for accurate diagnosis of many cancers; for example, the detection of ALK rearrangements in lymphoma and lung cancer helps clinicians provide more precise diagnosis and treatment. Fluorescence in situ hybridization (FISH) and immunohistochemistry (IHC) are two routine approaches used to detect ALK rearrangements. However, difficulties with acquisition of biopsy samples, high costs, and long waiting time for results negatively impact the application of these methods. A rapid and inexpensive alternative would be a useful complement to current ALK rearrangement detection. We identified a novel gene, *sweyjawbu*, from Affymetrix microarray studies. Its expression correlated strongly with ALK in an analysis of 1037 cancer cell lines (correlation coefficient = 0.92). By comparing *sweyjawbu* transcript levels, it was possible to discriminate 12 ALK rearrangement-positive lymphoma samples from 64 ALK rearrangement-negative lymphomas. Moreover, combining measurements of *sweyjawbu* expression and the ratio of the 5’ and 3’ portions of the ALK transcript provided even more accurate identification of ALK rearrangement-positive lymphomas. This novel approach is an excellent complement or alternative to existing FISH and IHC methodologies.

## INTRODUCTION

Lymphoma is one of ten most common malignancies worldwide, and chemotherapy is the backbone for its treatment. Highly aggressive relapsed and refractory lymphomas remain a major challenge for clinicians [1, 2]. Genetic alterations commonly cause constitutive activation of kinases in lymphomas. Anaplastic lymphoma kinase (ALK), also known as ALK tyrosine kinase receptor or CD246 (cluster of differentiation 246), is an enzyme encoded by the human ALK gene [3]. Functional chimeric proteins formed by fusing ALK with other proteins have found in many lymphoma subtypes [4], including anaplastic large-cell lymphoma (ALCL) and diffuse large B-cell lymphoma (DLBCL). With the development of precision medicine, detection of ALK rearrangements are now required in new diagnosis of ALCL and may also be considered in DLBCL [5]. Crizotinib, an inhibitor of tyrosine receptor kinases, including ALK and CD117, has been found to be effective against inflammatory myofibroblastic tumor or non-small cell lung cancer with ALK rearrangement [6]. Successful applications of the drug on the target protein/gene in ALK rearrangement- positive lymphoma have also been reported [7, 8]. FISH and/or IHC are currently used to characterize ALK rearrangement status. However, these techniques require tissue samples which are difficult to obtain from specific lymphatic disease patients.

The widely used Affymetrix U133 Plus 2.0 microarray allows us to test expression of thousands of genes simultaneously, including both coding and non-coding RNA. With this microarray, *Piva et al*. compared the expression profile from 16 ALCL samples with confirmed ALK rearrangement and 20 ALK rearrangement-negative ALCL samples and selected probe set 242964_at as one of the most significant differentially expressed genes, with a 47.58 fold change. Probe set 242964_at corresponds to *sweyjawbu* RNA, determined after aligning its probe sequences to an AceView RNA sequence collection database (www.ncbi.nlm.nih.gov/ieb/research/acembly) [9, 10]. We characterized the expression of *sweyjawbu* in a number of lymphoma cell lines in order to evaluate its usefulness as a predictor of ALK rearrangement status.

## RESULTS

### sweyjawbu expression is highly upregulated in ALK rearrangement-positive cell lines

**Figure 1:**
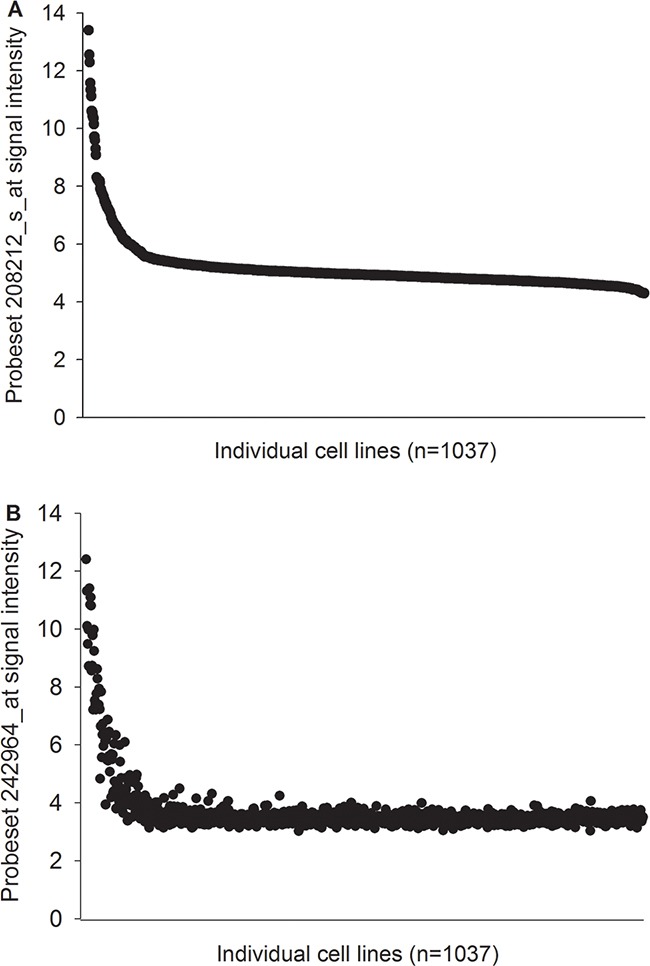
sweyjawbu expression is highly associated to ALK activated transcription Probe sets 208212_s_at (ALK) and 242964_at (*sweyjawbu*) signal intensities from 1037 different cell lines from Cancer Cell Line Encyclopedia (CCLE) database were plotted. Samples were arranged from highest to lowest by comparing the 208212_s_at probe set signal intensity. Those two probe sets’ expression were highly correlated.

The Cancer Cell Line Encyclopedia (CCLE) database provided analysis results of mRNA expression for 1037 cancer cell lines (www.broadinstitute.org/ccle) [9]. *sweyjawbu* RNA expression is associated with ALK gene amplification or translocation status in a number of different types of cancer cells. The correlation coefficient between signal intensity of probe sets 208212_s_at and 242964_at in 1037 cancer cells reached 0.92 (Figure 1).

**Figure 2:**
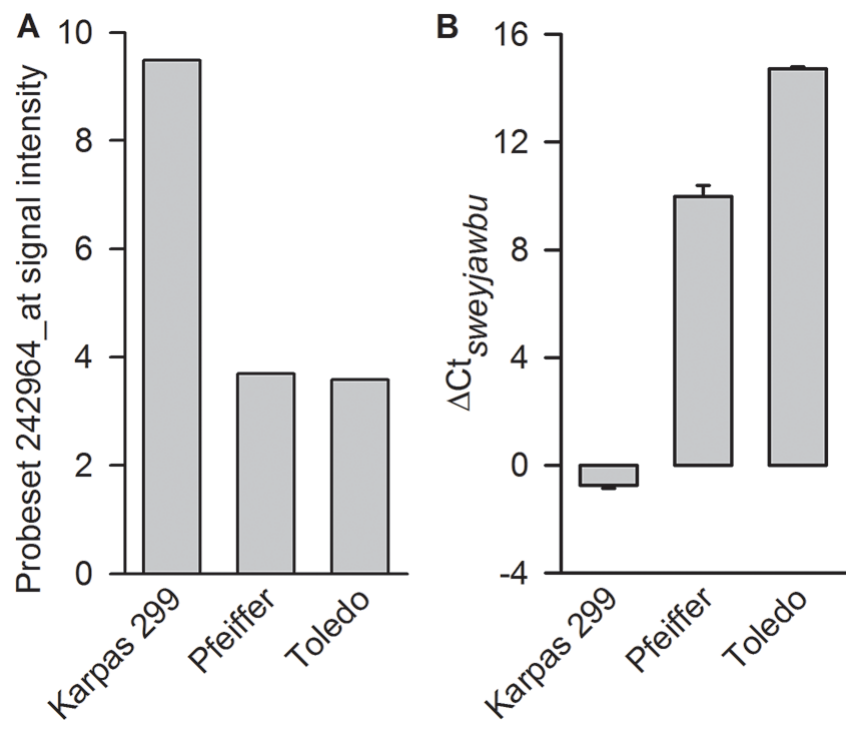
Differences were observed between ALK rearrangement-positive and ALK rearrangement-negative lymphoma cells **A**. Compare probe set 242964_at signal intensity. **B**. Compare the relative expression (ΔCt) value for *sweyjawbu* gene. Probe set 242964_at signal intensity was extracted from Cancer Cell Line Encyclopedia,www.broadinstitute.org/ccle. PCR experiments were performed in quadruplicate. There is no amplification for *sweyjawbu* gene in Toledo cell. Then the corresponding Ct value for *sweyjawbu* gene was set to 35 for result demonstration.

The signal intensities from three lymphoma cell lines were extracted from the dataset for demonstration. Probe set 242964_at exhibited positive expression only in ALK rearrangement-positive cells, but was below background level in ALK rearrangement-negative cells (Figure 2A). Real-time PCR experiments also indicated that there was more than one hundred fold difference between ALK rearrangement-positive cells and ALK rearrangement-negative cells in sweyjawbu RNA expression level (Figure 2B).

### PCR assay detects ALK rearrangement status in lymphomas

**Figure 3:**
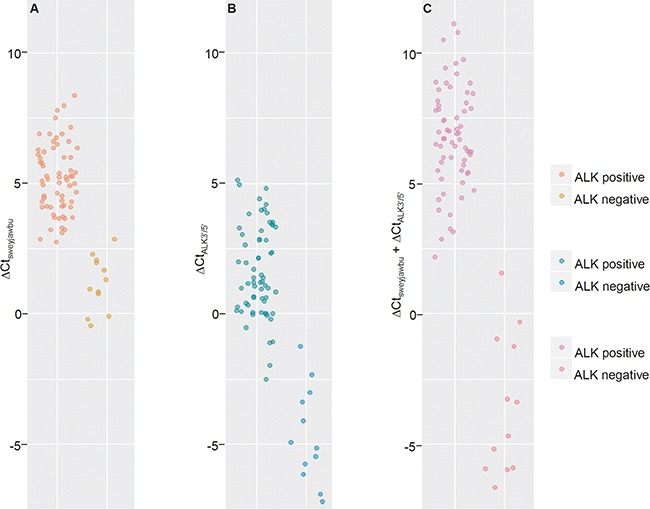
Differences were observed between ALK rearrangement-Positive lymphomas and ALK rearrangement-negative lymphomas **A**. Compare ΔCt value for *sweyjawbu* gene. **B**. Compare ΔCt value for 5’ portion and 3’ portion of ALK transcript. **C**. Compare ΔCt value for *sweyjawbu* gene + ΔCt value for 5’ portion and 3’ portion of ALK transcript. 13 ALCL tumors and 63 DLBCL tumors were included into this study. The 11 ALCL and 1 DLBCL tumors confirmed as ALK-positive with FISH or IHC, exhibited lower ΔCt value. Combination of ΔCt_sweyjawbu_ and ΔCt_ALK3’/5’_ could distinguish ALK rearrangement positive samples from ALK rearrangement-negative samples, despite a few misclassifications when used separately.

ALK rearrangement in lymphomas is relatively rare. There were 11 ALK rearrangement-positive ALCL patients and 1 ALK rearrangement positive DLBCL patient among the 13 ALCL and 63 DLBCL patients used in our experiment. Comparable results were observed from PCR assay and IHC or FISH tests, to determine ALK rearrangement status in lymphomas. *sweyjawbu* gene expression differed significantly between ALK rearrangement positive lymphomas and ALK rearrangement-negative lymphomas. *sweyjawbu* also improved the discriminative power of the 5’ and 3’ portion ratio of the ALK transcript (Figure 3; Supplementary Table 2).

### RNA in situ hybridization localizes sweyjawbu and ALK in lymphomas

**Figure 4:**
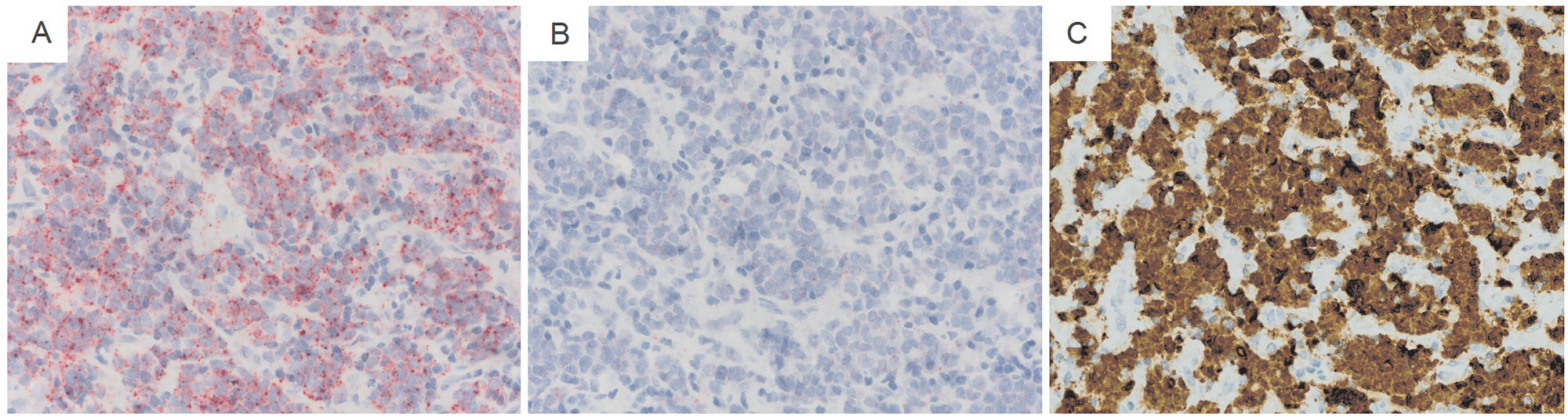
In situ hybridization to characterize ALK RNA and sweyjawbu RNA expression in lymphoma cells Intensive fast red Staining was observed in ALK rearrangement positive lymphoma cells. **A**. Section hybridized with probes target human ALK exon 19 to exon 29. **B**. Section hybridized with probes target human *sweyjawbu*. **C**. IHC was performed to determine ALK rearrangement status.

RNAscope technology provides a revolutionary RNA *in situ* hybridization method to characterize RNA expression in single cells. Significant ALK kinase domain RNA expression was observed in ALK rearrangement-positive lymphoma cells. Simultaneously, ALK rearrangement positive lymphoma cells also expressed *sweyjawbu* RNA. *In situ* RNA hybridization confirmed the association of *sweyjawbu* and ALK (Figure 4).

## DISCUSSION

With the rapid evolution of microarray technology over the last decade, multiple studies have characterized cancer cell lines and tumor tissue using standardized genome-wide microarray, and have generated large volumes of gene expression data. However, standard Affymetrix probe set annotation is at the gene level, and it has been reported that transcript-level annotation improves the interpretation of gene expression data [10]. In our experiment, we have aligned probe sequences from probe sets in traditional U133 Plus 2.0 gene expression arrays against the currently most comprehensive collection of transcript sequences from AceView database. Aid from this RNA level re-annotation makes using publicly available microarray gene expression databases an economical and efficient way to perform other data-mining studies. For example, we identified a novel, non-coding RNA linked to ALK activation which had been recorded but had not been detailed by previous studies [11, 12].

There are several commercial tests to detect ALK rearrangements or hybrids [13]. FISH is a relatively well-standardized but expensive method that requires biopsy. The interpretation of FISH results also requires specialized training due to considerable reader-related variability [14]. FISH results are affected by other factors such as long-duration specimen fixation, high temperature, and improper protein digestion. IHC is a relatively inexpensive method for diagnosing ALK rearranged lymphoma that, like FISH, requires biopsy sample. Variations in fusion partner may also influence protein levels and location [14]. Next generation sequencing provides the capability to detect multiple molecular alterations in a single assay. However, it may take several weeks to complete the experiment and data analysis [15] and the approach requires more technical manipulation, which might delay its clinical application.

In this study, a novel non-coding RNA *sweyjawbu* was identified from microarray datasets for prediction of ALK rearrangement. Previously, no report detailed the *sweyjawbu* gene, and its *in vivo* function is unknown yet. The ALK gene maps to chromosome 2, from 30144451 to 29415634 (NCBI 37, August 2010), on the reverse strand, while the *sweyjawbu* gene maps to chromosome 2, from 29414340 to 29413859, also on the reverse strand. There was only a 1294bp distance between the two genes on the chromosome. *sweyjawbu* expression differed distinctly between ALK rearrangement-positive cells and ALK rearrangement-negative cells. RNAscope technology indicated both ALK kinase domain RNA and sweyjawbu RNA were co-expressed in lymphoma cells. Future studies will be undertaken to understand whether the *sweyjawbu* and ALK genes are transcriptionally coupled neighboring genes [16].

Highly comparable results can be generated from microarray and qRT-PCR platform [17]. Based on this discovery, combined with other published known methods, we have designed a PCR assay to detect ALK RNA hybrid status in both ALCL and DLBCL patient tissue samples. A significant advantage of the PCR-based assay is that it can be easily used in tissues obtained from minimally-invasive biopsy such as fine needle aspiration (FNA) or Cerebro-Spinal Fluid (CSF), which may not provide tissue suitable for preparation of FFPE samples [18]. The assay also could be complementary to FISH and IHC, which might further improve the accuracy and convenience of diagnosis and treatment of ALK rearrangement-positive lymphomas, or even other lymphomas of unknown origin with rare ALK-positive condition. Moreover, the prototype assay could be transferred to the FilmArray platform [19], which is an FDA-cleared multiplex PCR system that integrates sample preparation, amplification, detection and analysis in an hour. In summary, the new essay has the potential to change current clinical practice.

## MATERIALS AND METHODS

### Cancer cell lines and clinical samples

ALK rearrangement-positive cell line Karpas 299 (ALCL), and two ALK rearrangement-negative cell lines, Pfeiffer (DLBCL) and Toledo (DLBCL) were utilized. Karpas 299 cell line from Sigma-Aldrich was maintained in RPMI 1640 medium supplemented with 20% Fetal Bovine Serum (FBS) and 2 mM L-glutamine. ATCC cell lines, Pfeiffer and Toledo, were cultured in RPMI-1640 medium supplemented with 10% FBS and 2 mM L-glutamine. Cells were cultured with 6cm dishes, and roughly 1 × 10^6^ cells were collected with RNAprotect cell reagent (Qiagen). After homogenizing the lysate with QIAshredder spin column (Qiagen), the RNAs were extracted with RNeasy Plus mini kit (Qiagen).

Fresh frozen lymphoid tissues were obtained from 3 ALCL and 63 DLBCL patients for analysis. In addition, 10 Formalin-fixed and paraffin-embedded (FFPE) tissues prepared in the last 2 years, from ALK rearrangement positive ALCL patients were used. The median age of patients was 55 years old. There were more male patients (71%) than female patients (29%). Despite 11 patients with unknown status, there are more early stage patients (63%) than advanced stage patients (37%). Written informed consent was obtained from all participants. The study was approved by the Ethics Committee of Fudan University Shanghai Cancer Center, China, and the procedures were conducted according to the principles expressed in the Declaration of Helsinki. Detailed clinical information is provided in Supplementary Table 2. Frozen tissue resected from lymphoma patients were homogenized in TRIZOL using mortar and pestle in the presence of liquid nitrogen. RNAs were extracted with RNAeasy Plus mini kit (Qiagen). FFPE tissues were deparaffinized with xylene and RNA was subsequently extracted with RecoverAll Total Nucleic Acid Isolation Kit (ThermoFisher Scientific).

The RNA quality was checked by electrophoresis of total RNA, followed by staining with ethidium bromide. The absorbance of each RNA at 260nm and 280nm were measured using a spectrophotometer for RNA quantity and purity.

### Primer design

The primer pairs were designed using the Beacon Designer software (Premier Biosoft, Palo Alto, CA). Primers’ sequences were compared with UCSC SNP database for known single-nucleotide polymorphism (SNP) information. The final concentration of each primer in the reaction tube was 0.3μM. Primer pairs with efficient amplification and single melt curves were kept for further analysis. Detailed primer sequences were provided in Supplementary Table 1.

### Real-time PCR detection

To detect templates from cell lines or tissue samples, cDNA was synthesized from 1μg RNA with QuantiTect Reverse Transcription kit (Qiagen). Quantitative real-time PCR detection was performed on an ABI 7900HT instrument using QuantiFast SYBR Green PCR kit (Qiagen). After initial activation for 5min at 95°C, two-step cycling was utilized for 40 cycles with 10s at 95°C and 30s at 60°C, followed by a melting curve analysis. Gene expression was normalized by TB and PGK genes in lymphomas [20].

### RNAscope RNA in situ hybridization study

Advanced Cell Diagnostics (ACD) Company introduced RNAscope technology for RNA *in situ* hybridization. Standard 35 ZZ probes targeting human ALK exon 19 to exon 29 were used to characterize ALK RNA 3’ end region. Custom designed 8 ZZ probes targeting *sweyjawbu* were used. Continuous FFPE sections were hybridized with dedicated probes and detected with fast red dye.

### Patients’ ALK rearrangement status confirmation

Selected patients’ ALK rearrangement status were checked either by FISH (Abbott), or IHC (VENTANA ALK (D5F3) CDx Assay, Roche) by the Department of Pathology, Fudan University Shanghai Cancer Center. FISH-positive cases were defined as two positive ALK rearrangement patterns. Positive cases were defined as more than 15% strand-break signals or isolated red signal signals in 50 tumor cells. IHC staining was scored by two pathologists as follows: 0 (no staining); 1+ (faint, cytoplasmic staining); 2+ (moderate, smooth cytoplasmic staining); 3+ (intense, granular cytoplasmic staining) in more than 10% of tumor cells. Anti-ALK IHC staining results were interpreted using a binary scoring system: positive (3+ or 2+) or negative (1+ or 0).

## SUPPLEMENTARY MATERIALS FIGURES AND TABLES




