# Supplementary material for: Sweyjawbu expression is a predictor of ALK rearrangement status in lymphoma
Source: Oncotarget. 2016 Dec 10;8(5):7914–20. doi: 10.18632/oncotarget.13851 (PMC5352370; doi:10.18632/oncotarget.13851)
Supplement: Supplementary file 1 [file oncotarget-08-7914-s001.pdf]

Sweyjawbu expression is a predictor of ALK rearrangement status in lymphoma

SUPPLEMENTARY TABLES

Supplementary Table 1: Primer sequences used in current study

	Primer 1	Primer 2
ALK (exon 24-25)	CCTGTGGCTGTCAGTATT	AGGTCAAGAGGCAGTTTC
ALK (exon 18)	GGGAAGTGAATATTAAGCATTATC	AGAAGCAGATGACCTTGT
sweyjawbu	CCTAAAGGATACTGACAGAGGAGTTG	GTGRTAGAAAGGCACAACCTGAAA
TBP	CAGTGAATCTTGGTTGTAA	TGGCTCTCTTATCCTCAT
PRKG1	TTTAACTGGGAAGGCTTA	AATTACTTGTGTCTGTGG

Supplementary Table 2: Lymphoma patients' sample characteristics

See Supplementary File 1
