# Supplementary material for: Sweyjawbu expression is a predictor of ALK rearrangement status in lymphoma
Source: Oncotarget. 2016 Dec 10;8(5):7914–20. doi: 10.18632/oncotarget.13851 (PMC5352370; doi:10.18632/oncotarget.13851)
Supplement: Supplementary file 2 [file oncotarget-08-7914-s002.doc]

**Table S2: Lymphoma patients’ sample characteristics**

| **Sample** | **Age** | **Gender** | **Tissue** | **Subtype** | **Stage**  **(Ann Arbor)** | **ALK (FISH)** | **ALK (IHC)** | **ALK (PCR)** |
| --- | --- | --- | --- | --- | --- | --- | --- | --- |
| SZ1 | 33 | Male | Lymphoma, lymphoid | ALCL | 3 | + | + | + |
| SZ2 | 38 | Male | Lymphoma, lymphoid | ALCL | 2 | + | + | + |
| SZ3 | 76 | Male | Lymphoma, lymphoid | ALCL | 4 | NA | + | + |
| SH2 | 51 | Female | Lymphoma, lymphoid | ALCL | 3 | NA | + | + |
| SH3 | 25 | Female | Lymphoma, lymphoid | ALCL | 3 | NA | + | + |
| SH4 | 60 | Male | Lymphoma, lymphoid | ALCL | 2 | NA | + | + |
| SH5 | 40 | Male | Lymphoma, lymphoid | ALCL | 1 | NA | + | + |
| SH1 | 65 | Female | Lymphoma, lymphoid | ALCL | 2 | + | + | + |
| FF1 | 61 | Female | Lymphoma, lymphoid | ALCL | 1 | + | + | + |
| FF2 | 45 | Male | Lymphoma, lymphoid | ALCL | 3 | + | + | + |
| A26 | 31 | Male | Lymphoma, lymphoid | ALCL | 2 | + | + | + |
| E43 | 59 | Male | Lymphoma, lymphoid | ALCL | 3 | NA | - | - |
| 011 | 42 | Male | Lymphoma, lymphoid | ALCL | 2 | NA | - | - |
| F63 | 62 | Female | Lymphoma, lymphoid | DLBCL | 2 | NA | + | + |
| A01 | 27 | Female | Lymphoma, lymphoid | DLBCL | 2 | NA | - | - |
| H77 | 53 | Male | Lymphoma, lymphoid | DLBCL | 2 | NA | - | - |
| A58 | 78 | Female | Lymphoma, lymphoid | DLBCL | 2 | NA | - | - |
| E34 | 45 | Male | Lymphoma, lymphoid | DLBCL | 1 | NA | - | - |
| H47 | 59 | Female | Lymphoma, lymphoid | DLBCL | 1 | NA | - | - |
| E63 | 56 | Female | Lymphoma, lymphoid | DLBCL | 1 | NA | - | - |
| H75 | 45 | Male | Lymphoma, lymphoid | DLBCL | 2 | NA | - | - |
| F57 | 41 | Male | Lymphoma, lymphoid | DLBCL | 1 | NA | - | - |
| G76 | 33 | Male | Lymphoma, lymphoid | DLBCL | 2 | NA | - | - |
| E72 | 59 | Male | Lymphoma, lymphoid | DLBCL | 2 | NA | - | - |
| F27 | 47 | Male | Lymphoma, lymphoid | DLBCL | 1 | NA | - | - |
| G45 | 78 | Female | Lymphoma, lymphoid | DLBCL | 3 | NA | - | - |
| E33 | 68 | Male | Lymphoma, lymphoid | DLBCL | NA | NA | - | - |
| F34 | 56 | Male | Lymphoma, lymphoid | DLBCL | 2 | NA | - | - |
| G32 | 62 | Male | Lymphoma, lymphoid | DLBCL | 2 | NA | - | - |
| A15 | 76 | Male | Lymphoma, lymphoid | DLBCL | NA | NA | - | - |
| 058 | 54 | Male | Lymphoma, lymphoid | DLBCL | 2 | NA | - | - |
| H48 | 51 | Female | Lymphoma, lymphoid | DLBCL | 3 | NA | - | - |
| DR15 | 64 | Male | Lymphoma, lymphoid | DLBCL | 3 | NA | - | - |
| A05 | 41 | Male | Lymphoma, lymphoid | DLBCL | 3 | NA | - | - |
| 046 | 56 | Male | Lymphoma, lymphoid | DLBCL | 2 | NA | - | - |
| DR34 | 49 | Male | Lymphoma, lymphoid | DLBCL | 4 | NA | - | - |
| DR80 | 71 | Male | Lymphoma, lymphoid | DLBCL | 1 | NA | - | - |
| A08 | 68 | Female | Lymphoma, lymphoid | DLBCL | 3 | NA | - | - |
| 054 | 62 | Male | Lymphoma, lymphoid | DLBCL | 3 | NA | - | - |
| DR35 | 58 | Male | Lymphoma, lymphoid | DLBCL | NA | NA | NA | - |
| DR17 | 50 | Male | Lymphoma, lymphoid | DLBCL | 1 | NA | - | - |
| DR81 | 69 | Female | Lymphoma, lymphoid | DLBCL | NA | NA | - | - |
| DR24 | 71 | Male | Lymphoma, lymphoid | DLBCL | 1 | NA | - | - |
| DR26 | 23 | Male | Lymphoma, lymphoid | DLBCL | 1 | NA | NA | - |
| DR27 | 37 | Male | Lymphoma, lymphoid | DLBCL | 4 | NA | - | - |
| DR18 | 39 | Female | Lymphoma, lymphoid | DLBCL | 1 | NA | - | - |
| DR9 | 51 | Male | Lymphoma, lymphoid | DLBCL | 1 | NA | - | - |
| A60 | 38 | Male | Lymphoma, lymphoid | DLBCL | 4 | NA | - | - |
| 059 | 39 | Female | Lymphoma, lymphoid | DLBCL | NA | NA | - | - |
| 014 | 73 | Female | Lymphoma, lymphoid | DLBCL | 2 | NA | - | - |
| DR28 | 64 | Male | Lymphoma, lymphoid | DLBCL | 1 | NA | - | - |
| DR10 | 69 | Male | Lymphoma, lymphoid | DLBCL | 4 | NA | - | - |
| DR74 | 74 | Male | Lymphoma, lymphoid | DLBCL | 3 | NA | - | - |
| 095 | 47 | Male | Lymphoma, lymphoid | DLBCL | NA | NA | - | - |
| DR29 | 62 | Male | Lymphoma, lymphoid | DLBCL | 4 | NA | - | - |
| DR20 | 56 | Female | Lymphoma, lymphoid | DLBCL | 1 | NA | - | - |
| DR2 | 47 | Male | Lymphoma, lymphoid | DLBCL | 3 | NA | NA | - |
| DR75 | 53 | Male | Lymphoma, lymphoid | DLBCL | 1 | NA | - | - |
| A31 | 76 | Female | Lymphoma, lymphoid | DLBCL | NA | NA | - | - |
| 096 | 18 | Male | Lymphoma, lymphoid | DLBCL | 2 | NA | - | - |
| DR39 | 73 | Male | Lymphoma, lymphoid | DLBCL | 4 | NA | - | - |
| DR30 | 47 | Male | Lymphoma, lymphoid | DLBCL | 2 | NA | - | - |
| DR21 | 48 | Male | Lymphoma, lymphoid | DLBCL | NA | NA | NA | - |
| DR3 | 46 | Male | Lymphoma, lymphoid | DLBCL | 3 | NA | NA | - |
| 099 | 33 | Male | Lymphoma, lymphoid | DLBCL | 2 | NA | - | - |
| 076 | 85 | Female | Lymphoma, lymphoid | DLBCL | 2 | NA | - | - |
| DR22 | 54 | Male | Lymphoma, lymphoid | DLBCL | NA | NA | - | - |
| DR13 | 60 | Male | Lymphoma, lymphoid | DLBCL | 3 | NA | NA | - |
| DR77 | 60 | Female | Lymphoma, lymphoid | DLBCL | 3 | NA | - | - |
| 077 | 61 | Female | Lymphoma, lymphoid | DLBCL | NA | NA | - | - |
| DR23 | 57 | Female | Lymphoma, lymphoid | DLBCL | 1 | NA | - | - |
| DR5 | 67 | Male | Lymphoma, lymphoid | DLBCL | 1 | NA | NA | - |
| DR69 | 52 | Male | Lymphoma, lymphoid | DLBCL | NA | NA | - | - |
| DR19 | 82 | Male | Lymphoma, lymphoid | DLBCL | 1 | NA | - | - |
| DR6 | 54 | Male | Lymphoma, lymphoid | DLBCL | 3 | NA | - | - |
| DR25 | 23 | Male | Lymphoma, lymphoid | DLBCL | 2 | NA | - | - |

NA : data not available.
